# Supplementary material for: Alpha-Tubulin Acetylation in Trypanosoma cruzi: A Dynamic Instability of Microtubules Is Required for Replication and Cell Cycle Progression
Source: Front Cell Infect Microbiol. 2021 Mar 11;11:642271. doi: 10.3389/fcimb.2021.642271 (PMC7991793; doi:10.3389/fcimb.2021.642271)
Supplement: Supplementary file 1 [file Image_1.pdf]

Supplementary Material

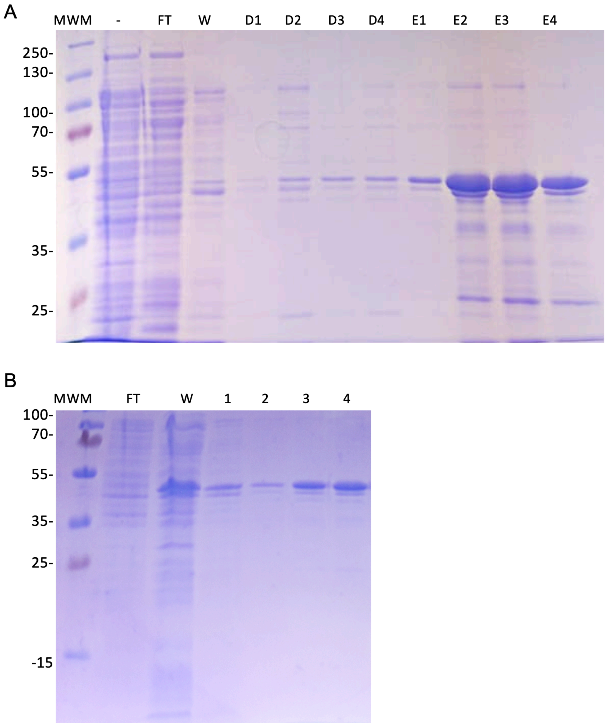

Supplementary Figure 1. Recombinant ATAT purifications. **(A)** Coomassie blue-stained SDS-PAGE of the 6xHis-tagged *Tc*ATAT purification from *E. coli* used for rabbit immunization and polyclonal antibodies production. MWM, molecular weight marker; (-), bacterial lysate uninduced with IPTG; FT, flowthrough obtained after incubating the induced bacterial lysate with the Ni^{+}-agarose resin; W, wash; D1-D4, elution with NaH_2PO_4 100 mM, Tris 10 mM, Urea 8 M pH 5.9; E1-4, elution with NaH_2PO_4 100 mM, Tris 10 mM, Urea 8 M pH 4.5. **(B)** Coomassie blue-stained SDS-PAGE of the HA-tag *Tc*ATAT purification from *T. cruzi* epimastigotes using an anti-HA resin. This protein was used for the autoacetylation assay. MWM, molecular, weight marker; FT, flowthrough obtained after incubating the resin with the epimastigotes lysate; W, wash; 1-4, elution with HA peptide 0.1 mg/mL.

```
Tb427_030013700     1 MTH-------NVMCDDVLPQLNL-PDGVTRWNANLLEE----ERRLRNSDGHA---DRII
LmjF.25.1150        1 MRRRPPQLTKTKLADEEVPELTLVPDGVSRWTGSDLDALLNAARRGGAEAAQQDLERKLC
TcCLB.467287.10     1 MSS-------TSQ-VALLPKLSL-PDGVTVWDGTALEY----ERRCNNVDEHA---VHLM

Tb427_030013700    46 LTINTLGKRSKEAQSLNTILTSVPRLRENRDARLYLLCHGGRGVGILKIGVKRLFVVPPS
LmjF.25.1150       61 RTIDILGARSQQAQEINAVLTSVARLRENSTFRLYLLTQNHRGVGILKVGVKKLFVTHPV
TcCLB.467287.10    45 QTINILGIRSKEAQCLNTVLTSVARLRENRQARVYLLCQDGYGVGILKMGVKKLFVTHPS

Tb427_030013700   106 HAGLMEIEPVCVLDFFVDTSNQRQGYGKILFEHMLAFERLS-PGDVAIDRPSVKFLAFLR
LmjF.25.1150      121 TCGLVEVDPLCVLDFYVDESCQRQGYGKMLYSHMLKAEHVSRPEVLAIDRPSNKLLGFLR
TcCLB.467287.10   105 YSSLVEIDPLCVLDFFVDTSFQRKGFGKTLFDAMLLNEGLN-PGEVAIDRPSVKFLAFLQ

Tb427_030013700   165 KHYGLVEYTPQSNNFVVFHKYFERHQQQRRGVGGSGRSG--------YQHCNETTT---Q
LmjF.25.1150      181 KHYGLAAYTPQVNNFVVFHSFFDHTTVSERGKLL--------------------------
TcCLB.467287.10   164 KYYGLVEYTPQSNNFVVFHRYFDKWQPQR-GKGHWGGNAVPTRSLVRPQNCLRVYPKYQS

Tb427_030013700   214 QLGTQSGLLEDINQTHPAPSYALRGVVMGHTGPPLDLTNVTQQQKPYHQPFATGRKTSYE
LmjF.25.1150      215 --------------RAPSP---------------A-------------------------
TcCLB.467287.10   223 TTGPNNNFEEDATHRTPPP---------------PPLPPPLVPQGSVTSP-GVGKKTAYE

Tb427_030013700   274 LQYERYLQSQNCR--PTGNAGYGGGNGPASSAEVRATNCQARRRTSPTRSGVPYNIINGS
LmjF.25.1150      221 --------------------R--ASPFPSSANATVAIGGAKAKNWTP-------------
TcCLB.467287.10   267 LQYEEYLREQAYRRRQGGDPRLQPVPNPVSSSEIVAASCGARRRMSPTRSGVQYNIISGT
```

Supplementary Figure 2. Multiple sequence alignment of *Tc*ATAT (TcCLB.467287.10) and its homologues in *Trypanosoma brucei* (Tb427_030013700) and *Leishmania mayor* (LmjF.25.1150) using T-coffee server and colored with Boxshade. The acetyltransferase domain is boxed in green.

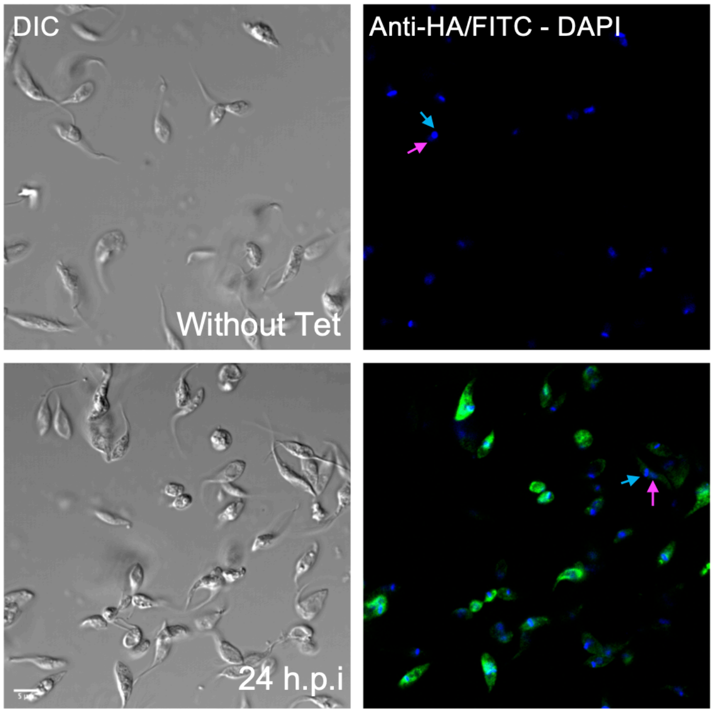

Supplementary Figure 3. Immunolocalization of ATAT-HA with rat monoclonal anti-HA antibodies in Dm28c p*Tc*INDEX-GW ATAT-HA epimastigotes uninduced (Without tet) and induced with 0.5 µg/ml tetracycline 24 hours post-induction (h.p.i). Bar: 5 µm. DAPI was used as nucleus and kinetoplast marker. The light blue arrow indicates the kinetoplast and the pink arrow indicates the nucleus.

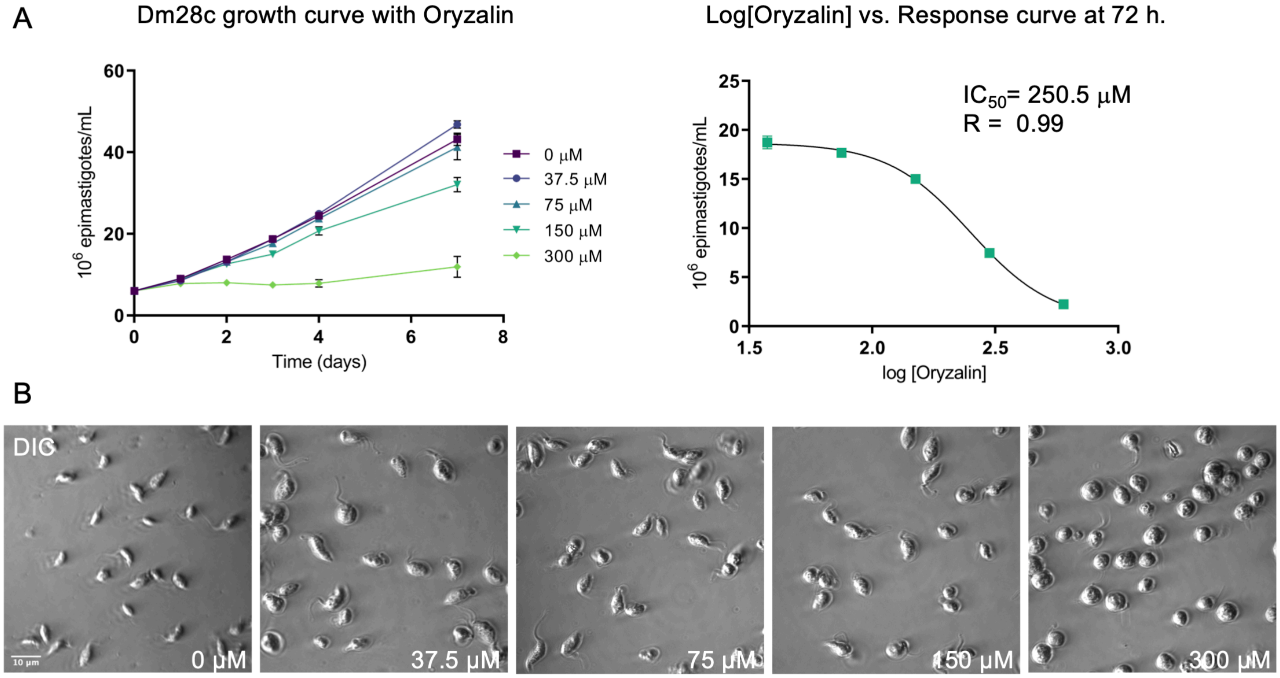

Supplementary Figure 4. (A) Growth curve of Dm28c epimastigotes in the presence of increasing concentrations of Oryzalin (0-300 µM) (left panel) and number of parasites *versus* the log [Oryzalin]

at 72 h (right panel). The latter plot was fitted with the non-parametric regression log(inhibitor) vs. response -Variable slope (four parameters) in GraphPad Prism version 8.0 to obtain the IC_{50} value and the R^2 of the fit. **(B)** DIC images of the morphological changes in Dm28c epimastigotes with different concentrations of Oryzalin at 72 hours. Bar = 10 μm.

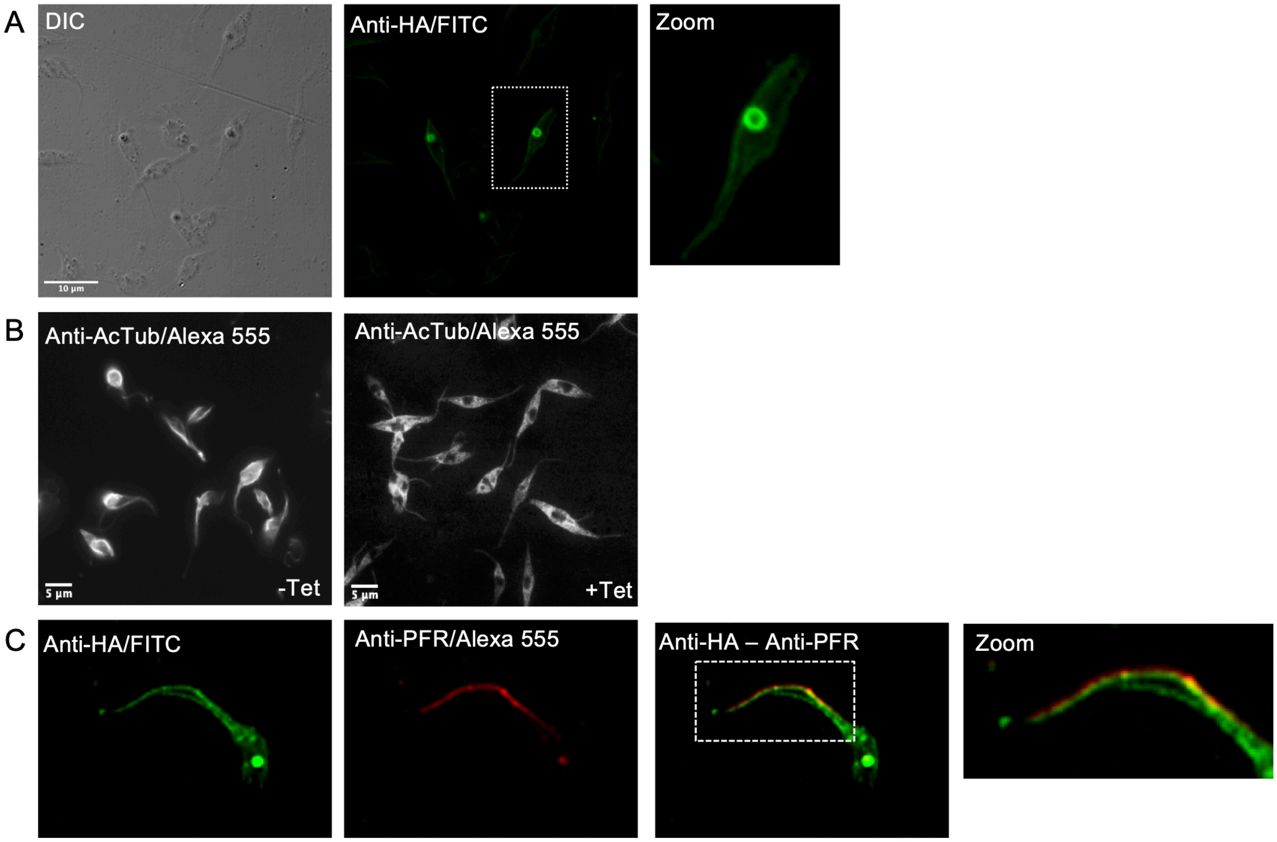

Supplementary Figure 5. (A) Immunolocalization of ATAT-HA with rat monoclonal anti-HA antibodies in isolated cytoskeletons of Dm28c pTcINDEX-GW ATAT-HA epimastigotes induced with 0.5 μg/ml tetracycline for 24 h **(B)** Immunolocalization of acetylated a-tubulin with mouse monoclonal anti-acetylated a-tubulin (anti-AcTub) in isolated cytoskeletons of Dm28c pTcINDEXGW ATAT-HA epimastigotes cytoskeletons uninduced (-Tet) and induced with 0.5 μg/ml tetracycline for 24 h (+ Tet). **(C)** Immunolocalization of ATAT-HA with rat monoclonal anti-HA antibodies and mouse 5 polyclonal anti-paraflagellar rod 2 from *T. cruzi* (anti-PFR) in isolated cytoskeletons of Dm28c pTcINDEX-GW ATAT-HA epimastigotes induced with 0.5 μg/ml tetracycline for 24 h.

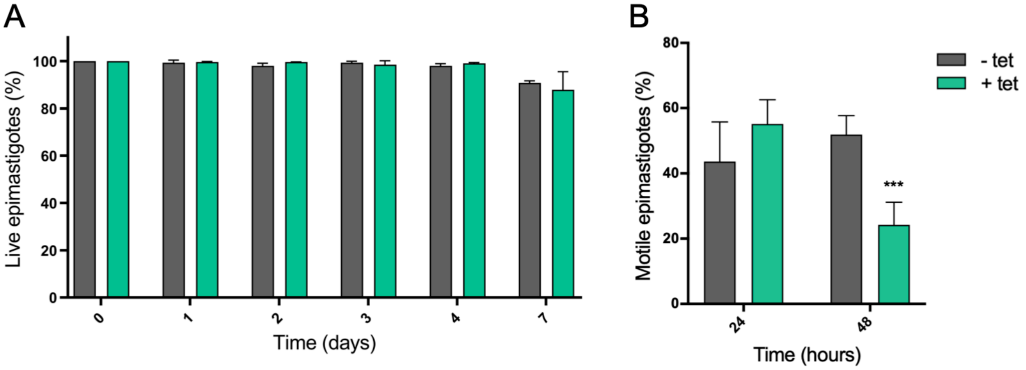

Supplementary Figure 6. (A) Viability (% of live epimastigotes) of Dm28c p*Tc*INDEX-GW ATAT-HA epimastigotes uninduced (grey bar, -tet) and induced with 0.5 µg/ml tetracycline (green bar, +tet) determined by counting live cells with a hematocytometer using Erythrosin B staining for 7 days. **(B)** Epimastigotes movements were examined using the computer-assisted semen analysis (CASA) system (Microptic, SCA evolution). The mean path velocity (VAP, µm/sec) was plotted at 24 and 48 h.p.i. without (grey bar, -tet) and with 0.5 µg/ml tetracycline (green bar, +tet). Experiments were performed in triplicates.

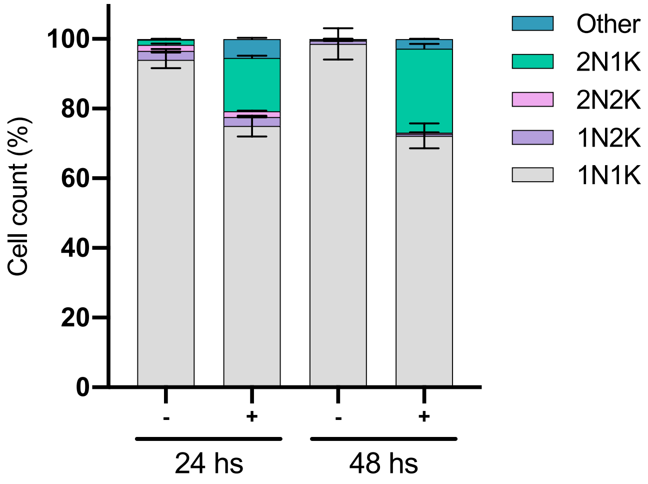

Supplementary Figure 7. Nucleus/Kinetoplast content (N/K) of Dm28c p*Tc*INDEX-GW ATAT-HA epimastigotes cultures in the absence (-) or presence (+) of 0.5 µg/ml tetracycline at different time points (24 and 48 hours). Data from three independent experiments were considered in the analysis (n = 300 cells for each column).
